# Case conferences between general practitioners and specialist teams to plan end of life care of people with end stage heart failure and lung disease: an exploratory pilot study

**DOI:** 10.1186/1472-684X-13-24

**Published:** 2014-05-05

**Authors:** Geoffrey Mitchell, Jianzhen Zhang, Letitia Burridge, Hugh Senior, Elizabeth Miller, Sharleen Young, Maria Donald, Claire Jackson

**Affiliations:** 1 School of Medicine, University of Queensland, Salisbury Road, Ipswich 4305, Australia; 2 Discipline of General Practice, School of Medicine, University of Queensland, Herston Rd, Herston 4006, Australia

## Abstract

**Background:**

Most people die of non-malignant disease, but most patients of specialist palliative care services have cancer. Adequate end of life care for people with non-malignant disease requires acknowledgement of their limited prognosis and appropriate care planning. Case conferences between specialist palliative care services and GPs improve outcomes in cancer-based populations. We report a pilot study of case conferences between the patient’s GP and specialist staff to facilitate care planning for people with end stage heart failure or non-malignant lung disease in a regional health service in Queensland Australia.

**Methods:**

Single face to face case conferences about patients with a primary diagnosis of advanced heart failure or respiratory failure from non-malignant disease were conducted between a palliative care consultant, a case management nurse and the patient’s GP. Annualised rates of service utilisation (emergency department [ED] presentations, ED discharges back to home, hospital admissions, and admission length of stay) before and after case conference were calculated. Content and counts of case conference recommendations, and the rate of adherence to recommendations were also assessed. A process evaluation of case conferences was undertaken.

**Results:**

Twenty-three case conferences involving 21 GPs were conducted between November 2011 and November 2012. One GP refused to participate. Ten patients died, three at home. Of 82 management recommendations made, 55 (67%) were enacted. ED admissions fell from 13.9 per annum (pa) to 2.1 (difference 11.8, 95% CI 2.2-21.3, p = 0.001); ED admissions leading to discharge home from 3.9 to 0.4 pa (difference 3.5, 95% CI -0.4-7.5, p = 0.05); hospital admissions from 11.4 to 3.5 pa (difference 7.9, 95% CI 2.2-13.7, p = 0.002); and length of stay from 7.0 to 3.7 days (difference 3.4, 95% CI 0.9-5.8, p = 0.007). Participating health professionals were enthusiastic about the process.

**Conclusions:**

This pilot is the initial step in the development and testing of a complex intervention based on a model of integrated care. A single case conference involving the patient’s heart or lung failure team is associated with significant reductions in service utilization, apparently by improving case coordination, enhancing symptom management and assessing and managing carer needs. A randomized controlled trial is being developed.

**Trial registration:**

Australian and New Zealand Controlled Trials Register ACTRN12613001377729: Registered 16/12/2013.

## Background

In common with most developed countries, Australia faces a rapid increase in the proportion of its population who are old or very old. It is estimated that 22% of Australians will be over 65 years, and 5% over 80 in 2061 [1]. It follows that the numbers of people at the end of life will grow rapidly as well. Most people who die have a period of inexorable deterioration that is predictable. However, the actual time of death will be dependent on the nature of the condition, and is much harder to predict [2]. This makes service planning very difficult indeed.

Over 80% of Australian specialist palliative care services are provided to cancer patients [3]. However, most patients die from non-malignant conditions [4] and will be under the care of other health professionals, both specialists and generalists. While most general practitioners (GPs) have little palliative care training, they are experienced in caring for people with advanced chronic disease. Further, many of these patients are cared for by system-based specialists, or those with more multifaceted expertise, such as gerontologists. The current system is set up to react to health challenges, and this consumes a large proportion of the health budget. Less attention is paid to proactive care planning [5].

A move to alter the paradigm of end of life care from reaction to a more proactive approach has evolved in the last decade [6-8]. This approach essentially assumes that the end of life can be anticipated. It should therefore be possible to anticipate the nature of potential problems, and put measures in place to ameliorate or even prevent such problems. These assumptions have led to the development of a sophisticated program of proactive case identification and anticipatory care planning, initially developed in English general practice, but moving into aged care and acute hospital settings [9-11].

As these concepts have been explored further, it is clear that implementing case finding and care planning is difficult. The reasons for this relate to the health system within which practitioners must operate [5]; the pressures that reduce their ability to set time aside to find cases and to consider the care plan, and a natural reluctance to acknowledge the impending death of a patient [8].

The use of single case conferences between specialist palliative care teams and a person’s GP has been tested for people already referred to palliative care (therefore predominantly suffering cancer), with demonstrated improvement in quality of life in the last month of life [12], retention of function [13], and a reduction in the number of hospital visits [13]. It is not known whether similar impacts will occur if case conferences are conducted for people with end stage non-malignant disease with a less predictable disease course.

We report a pilot study of case conferences between a specialist palliative care physician, a visiting nurse specialist and the patient’s GP for non-malignant patients identified as approaching the end of life.

## Methods

### Setting

This study was conducted in the West Moreton Health and Hospital Service (WMHHS) District, Queensland, Australia. It is a district of approximately 240,000 people, serviced by a district general hospital (Ipswich Hospital) and four smaller rural hospitals. The Heart Failure and Lung Health services work in conjunction with hospital-based specialist services, and run outreach specialist nursing services to provide case management. General medical care is provided by the patient’s GP. Coordination of these two services is made difficult by the organization of the health system: Australian hospitals are a State government responsibility, and community-based general practice is a Federal responsibility. A discharge summary is provided after ED and inpatient admissions, and case coordinating nurses liaise with GPs as required. There is no shared health record. However, funding for GPs and specialist physicians is available for care coordination and care planning activities, including case conferences, between health professionals through Medicare, the national health insurance scheme. WMHHS nurses are salaried and liaison with the GP is part of their normal work practice.

### Study objectives

The overall project aim is to assess the effectiveness of case conferences between specialist teams and GP in improving patient outcomes for people with end stage heart failure or lung disease. The objectives of this pilot study were to:

1. Provide an estimate of the effect of the intervention on service utilization, and

2. Demonstrate the feasibility and acceptability of the process of case conferences.

We used a pre-post design, and included patients who had a case conference in a twelve month period from November 2011. Cases were included if there was at least a three month follow-up period.

### Process development

The case conference process was developed in conjunction with WMHHS Heart Failure and Lung Health teams and palliative care staff over a six month period. The outcomes of this process were a document for nurses to provide a preliminary report of key palliative care issues for discussion, and a reporting document/care plan described below.

### Participants and case conference process

**Figure 1:**
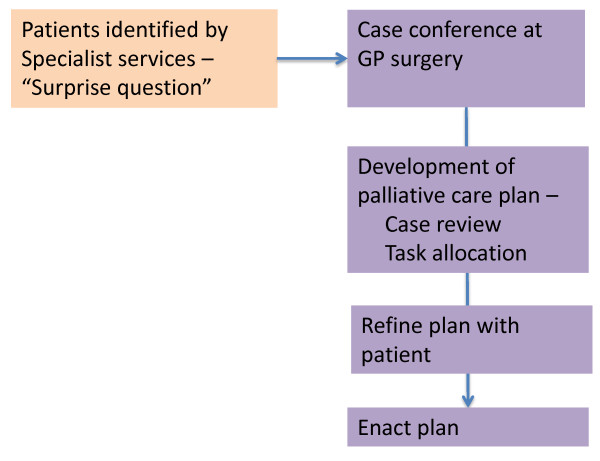
Flow chart of case conference intervention.

Patients already registered with either the Heart Failure Service or the Lung Health Service were identified by clinic staff as being at risk of dying in the foreseeable future, using the “surprise question” (Would you be surprised if this patient died within the next twelve months?) [14]. After acquiring informed consent from the patient, a case conference was arranged. This took place at the GP’s surgery between the GP, a palliative care physician and the case management nurse caring for the patient. The patient was not involved in the case conference, but prior discussions between the nurse, patient and carer highlighted issues of importance to them that would be raised at the meeting. The nurse also provided a written summary comprising the key diagnoses and a summary of the palliative problems as seen by the nurse and patient/carer, to the team members prior to the case conference. The palliative care physician (GM) did not see the patient prior to the case conference – rather he facilitated a case review between the clinicians actively involved in the case, and provided clinical advice as needed (Figure 1).

The content of the case conference was guided by a semi-structured schedule, based on the PEPSI COLA mnemonic used in the Gold Standards Framework care planning documents (Table 1) [9]. This ensured that the full range of issues likely to be of concern in a palliative setting was considered. A needs-based care plan was subsequently developed which identified possible actions, and who was responsible for each. Emphasis was placed on ongoing communication between the nurse and GP, after hours emergency plans, including educating the primary carer on how to deal with common anticipated problems, and systematically addressing the needs and concerns of primary carers using a modified carer needs checklist [15]. While both GPs and nurses followed up with the patient and carer, the nurse reviewed/explained the proposed plan and negotiated any changes to the plan.

**Table 1:** **The PEPSI COLA structure of palliative care health plans**[9]

**Domain**	**Issues to consider**	**Domain**	**Issues to consider**
**P**hysical	Symptom control	**C**ontrol	Choice, dignity
	Medication – regular and as needed		Treatment options/Management Plan
	Compliance/stopping non-essentials		Advance directive
	Complementary therapies		Place of death
**E**motional	Understanding expectations	**O**ut of hours/emergency	Continuity
	Depression and adjustment		Provision of out of hours care to patients/carers
	Fears/Security		
	Relationships		Carer support
			Medical support
			Drugs and equipment
**P**ersonal	Spiritual/religious needs	**L**ate	End of life/Terminal care
	Inner journey		Stopped non-urgent treatment
	Quality of life		Patient and family aware
	Patient/carer agenda		Comfort measures/Spiritual care
			Rattle, agitation
**S**ocial Support	Benefits/Financial	**A**fterwards	Bereavement follow-up/others informed
	Care for carers		
	Practical support		Family support
			Assessment/Audit
			Support team
**I**nformation/communication	Within Team		
	Between professionals		
	To and from patient		
	To and from carers		

### Study measures and data analysis

For patients with at least three months follow-up (to death or to census date), we collected health service utilisation data from health service records and GP records for up to twelve months follow-up. Case conferences were conducted between November 2011 and November 2012, with health service utilization data being collected between February and March 2013. Study outcomes included the number of ED visits, the number of ED visits not resulting in hospitalisation, the number of inpatient admissions and the length of inpatient stay. An inpatient admission was defined as an overnight stay in hospital, and included the short stay unit attached to the ED department. We compared up to a year of care after the case conference, with up to a year prior to the case conference. As different periods of time were analysed for each patient, service utilisation data were standardised to rates per annum.

We compared the pre- and post- case conference service utilisation using Wilcoxon signed rank test due to non-normally distributed data. Mean or median and 95% confidence intervals (CIs) were calculated. A statistically significant difference of the mean ranks was set at p ≤ 0.05. SPSS Version 21 [16] was used for the data analysis. The number of recommendations in the care plan, and the rate of uptake of the recommendations arising from the case conference were also assessed using basic descriptive statistics. After each case conference, we asked all participating health practitioners to complete a short questionnaire where they described their observations of the process and usefulness of the exercise. Patients and carers did not participate in this evaluation.

This study was approved by the WMHHS Ethics Review board, and the University of Queensland Behavioural and Social Sciences Ethical Review Committee. Informed consent was obtained from all participants. The trial has been registered with the Australian and New Zealand Clinical trials registry: ACTRN12613001377729 – registered 16/12/2013.

## Results

Twenty-one GPs participated, with two GPs having two cases each; additionally, one GP refused to participate (believing that case conferences would be a waste of time), resulting in a total of 23 completed case conferences. Eighteen of the case conferences were conducted for patients with heart failure patients and five for those with advanced lung disease. Of these, 11 (47.8%) were female and the median age was 74 (range 61–89). Ten patients died, of whom three died at home. The median survival rate for these patients was 142 days (range 48–279) after the case conference.

### Service utilisation

Table 2 shows service utilisation data. There were statistically significant reductions in rates of ED visits, numbers of hospital admissions and length of stay, and numbers of ED visits not resulting in admission. These analyses were repeated excluding the data for one outlier for service utilisation, and the results were similar. In order to identify a potential learning effect for the nursing staff, we compared the service utilization rates of the first eleven case conferences (December 2011-July 2012) with the second twelve case conferences (August 2012-November 2012), and there were no statistically significant differences between these groups.

**Table 2:** Rates of service utilisation before and after case conferences

	**Full results**				**Excluding service utilisation outlier**			
	**Pre CC**	**Post CC**	**Difference (95% CI)**	**P**	**Pre CC**	**Post CC**	**Difference (95% CI)**	**P**
ED admissions (annualised number)	13.9	2.1	11.8 (2.2 – 21.3)	0.001	9.7	1.7	8.0 (2.2 – 13.8)	0.001
ED admissions not leading to hospital admission (annualised number)	3.9	0.4	3.5 (−0.4 – 7.5)	0.05	2.3	0.5	1.9 (−0.2 – 3.9)	0.09
Number of hospital admissions (annualised number)	11.4	3.5	7.9 (2.2 – 13.7)	0.002	9.1	3.0	6.1 (1.5 – 10.6)	0.003
Length of stay (days)	7.0	3.7	3.4 (0.9 – 5.8)	0.007	6.9	3.4	3.5 (0.9 – 6.0)	0.009

### Case conference recommendations

We examined the recommendations, and those recommendations that were actioned, for the whole group, as well as for those died and those who did not die. Eighty-two recommendations were generated from the 23 case conferences (Table 3). Of these, 55 had been enacted at the time of the data collection. Most recommendations arose from the physical, social support, emotional and control domains of the PEPSI COLA derived plan. More actions related to physical symptoms and control (particularly ensuring advance directives were in place) were made for people who died than those who did not, and more recommendations led to actions. There was complete uptake of recommendations for emotional issues in both groups. These recommendations included applying a carer needs checklist [15] and referrals to psychologists or social workers. All were actioned by the specialist team. All recommendations relating to communication between the GP and members of the specialist team were put in place for those who did not die, but not for all of those who did die. Overall, there was no difference in the proportion of recommendations that led to action in either group.

**Table 3:** Number of recommendations arising from case conferences

	**Total patients in sample (n = 23)**		**Patients who did not die (n = 13)**		**Patients who died (n = 10)**		
Domain*	**Number of recommendations**	**Number actioned (%)**	**Number of recommendations**	**Number actioned (%)**	**Number of recommendations**	**Number actioned (%)**	P^**^
Physical	24	15 (62.5%)	7	3 (43%)	17	12 (71%)	<0.001
Emotional	11	11 (100%)	7	7 (100%)	4	4 (100.0%)	N/A
Personal	3	1 (33.3%)	3	1 (33%)	0	0 (N/A^***^)	0.083
Social Support	12	7 (58.3%)	7	4 (57%)	5	3 (60.0%)	0.445
Information/communication	10	7 (70.0%)	5	5 (100%)	5	2 (40.0%)	N/A
Control	11	7 (63.6%)	7	3 (43%)	4	4 (100.0%)	0.037
Out of hours/Emergency	5	3 (60.0%)	1	1 (100%)	4	2 (50%)	N/A
Late	4	2 (50.0%)	1	0 (0%)	3	2 (67%)	N/A
Afterwards	2	2 (100%)	0	0 (N/A)	2	2 (100%)	N/A
**Totals**	**82**	**55 (67.0%)**	**38**	**24 (63%)**	**44**	**31 (71%)**	**0.146**

^*^For descriptions of each domains’ content, see Table 1.

^**^χ^2^ Test or Fishers Exact test if n < 5 in any array.

^***^N/A no calculation possible if value = 0 in the denominator of a pair.

### Health professional feedback on case conferences

Most health professionals appreciated the case conferences. The majority of GPs felt it was a good use of their time, although some thought they were time-inefficient, particularly in the first few that were conducted. All case management nurses found great value in them. In particular, they allowed the GPs to meet face to face with them and to be confident that the nurses were competent. This facilitated easier subsequent communication than before, when GPs were more reluctant to accept or return phone calls. They also reported that the patients and carers were pleased with the process and the subsequent plan, and felt their concerns were being addressed. Finally, nurses reported that over the course of the pilot, their knowledge and skills increased and their normal practice changed as a result of repeated exposure to the palliative care skills discussed during the case conferences.

## Discussion

Interventions such as the one described above are complex and require complex trial design [17]. This intervention includes pre-conference assessment by the nurse and GP, as well as the case conference itself. The UK Medical Research Council describes a development – evaluation – implementation process for testing complex interventions [18]. This research has focused on the first two phases of intervention development and evaluation. Further development, including the addition of formal evaluation of patient and carer outcomes is underway and will be reported at a later stage. To obtain more valid and generalisable data, we aim to conduct a randomised controlled trial of the case conference process.

**Figure 2:**
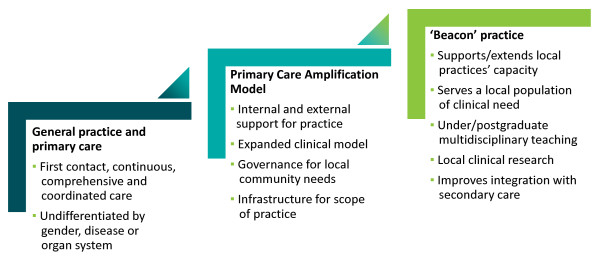
**The Beacon Practice model of care for complex conditions **[20]**.**

This paper describes the development and impact of case conferences between primary care and specialist public sector-based professionals involved in the care of people with end stage non-malignant disease. It is part of a broader research agenda exploring the interface between specialist and primary care, and uses the Beacon Practice model of such care as its theoretical framework [19,20]. (Figure 2) In its usual format this comprises community-based multidisciplinary clinics where GPs with a special interest (GPwSIs) in the index condition, a medical consultant, and nursing and allied health staff work together to manage complex medical problems [21]. GPwSIs undertake advanced training in the condition prior to working in the clinic. The model was first tested in complex diabetes care with promising results, in clinical outcomes, service efficiency and patient satisfaction [22]. A formal randomised controlled trial (RCT) is underway [23].

The adaptation of this model to non-malignant “end of life” care assumes that the GPwSI is the patient’s own GP due to their expert knowledge of *that patient*, rather than of a particular clinical condition. The extra training they receive occurs during the case conference. If the model becomes standard practice, their skill level in end of life care should increase with case conferences for more patients [24]. It was obvious to the palliative care physician in the study (GM) that, in nearly all cases, the GP’s knowledge of the patient’s condition and the skill demonstrated in managing the elements of the case was of a high standard, and the case conference added specific palliative care interventions for symptoms such as breathlessness and pain, as well as improved case coordination.

The key process outcome of a case conference, which is not present in normal care, is the systematic consideration of all aspects of end of life care. Not all elements will be relevant to each case, but all are at least considered. Further, attention is given to task allocation, so that the involved clinicians are aware of their own and their colleagues’ responsibilities towards the patient. This addresses the common problem of specialists and generalists operating in silos with each patient, so that some issues are covered by both, and some are missed because they are thought to be the other party’s responsibility.

A major contributor to the success of this project was that the delivery model was deliberately designed to encourage GP participation. Previous studies show the importance of adapting the process to the work practices of the participants involved [25,26]. In this research, we took the case conference to the GP. The work practices of GPs make it almost impossible to participate in multidisciplinary case conferences if they are required to physically attend off-site specialist team meetings. This process had benefits for the specialist nursing staff by establishing a relationship between them. This made communication after the case conference much easier than it had been before.

We have demonstrated major improvements in service utilisation in this small pilot, which we hypothesise arose because of this attention to detail and care coordination. The data on ED visits not resulting in admission is probably a proxy measure of the degree to which patients and carers are able to care for complications themselves, rather than use an ED visit as a means of managing problems not possible to be managed at home. This statistic fell by a factor of ten, but was influenced by a service utilisation outlier.

The proportion of recommendations enacted was somewhat low. The reason for this is not clear as there are non-significant differences in the proportion of uptake of recommendations for those who died and did not die. The case conference participants demonstrated priorities which depended depending on the closeness to death of the patient. This phenomenon does need further investigation.

There are limitations to this study. It is a small pre-post pilot study conducted by one service and one palliative care consultant. As there is no prospective control group, it is possible that the changes noted may have been caused by some factor other than the case conference. While there was probably an improvement in nursing care over the course of the pilot, we have shown this did not influence service utilisation data. Further, the number of patients who died is probably an underestimate of that which will occur in a prospective trial with a full twelve month follow-up, as the recruitment period was only fifteen months.

We took the approach that the pilot should be an efficacy trial, where an attempt was made to make conditions for the intervention as ideal as possible. There are inherent inefficiencies in conducting case conferences at the GP surgery. However, we demonstrated overwhelming support for the intervention from GPs and specialist nursing staff. An economic analysis of the cost benefit of this service model will be conducted as part of further studies. Further, we have begun to test the use of videoconferencing to improve efficiency of the process. This is particularly important in the Australian context, where time and distance are major impediments to service delivery. The Australian government has supported videoconferences in rural and remote areas and aged care facilities, with over 77,000 telehealth services offered to 33,000 patients by 7,700 practitioners in eighteen months [27]. However, urban GPs can also be isolated and video-conferences may work in this context as well.

## Conclusion

Case conferences for people with non-malignant life-limiting conditions are associated with significant reductions in health service utilisation. This form of communication has high uptake by GPs, and, while time consuming, may be cost effective. Further development of the process of data collection for patient outcome assessment is underway and a prospective RCT will be conducted in due course.

## Competing interests

The authors declare that they have no competing interests.

## Authors’ contributions

GM conceived study, oversaw trial design, overall responsibility for trial conduct, data interpretation, wrote main draft of the paper. JZ developed quantitative analysis protocol, conducted statistical analysis, wrote quantitative analysis section. LB developed qualitative analysis protocol. HS trial management, conceived health service utilisation data analysis protocol. EM assisted in trial development, day to day conduct of trial and data acquisition. SY day to day conduct of trial and data acquisition. MD co-developed trial protocol. CJ was principal researcher of Beacon Practice Model suite of projects. All authors contributed to and approved final version of paper.

## Pre-publication history

The pre-publication history for this paper can be accessed here:

http://www.biomedcentral.com/1472-684X/13/24/prepub
